# The Isolation of Orientia tsutsugamushi and Rickettsia typhi from Human Blood through Mammalian Cell Culture: a Descriptive Series of 3,227 Samples and Outcomes in the Lao People’s Democratic Republic

**DOI:** 10.1128/JCM.01553-20

**Published:** 2020-11-18

**Authors:** Damien K. Ming, Vanheuang Phommadeechack, Phonepasith Panyanivong, Davanh Sengdatka, Weerawat Phuklia, Vilada Chansamouth, Tamalee Roberts, Stuart D. Blacksell, Paul N. Newton, Matthew T. Robinson

**Affiliations:** a Faculty of Medicine, Imperial College, London, United Kingdom; b Lao-Oxford-Mahosot Hospital-Wellcome Trust Research Unit (LOMWRU), Microbiology Laboratory, Mahosot Hospital, Vientiane, Lao People’s Democratic Republic; c Mahidol Oxford Tropical Medicine Research Unit (MORU), Bangkok, Thailand; d Centre for Tropical Medicine & Global Health, Nuffield Department of Medicine, University of Oxford, Oxford, United Kingdom; Mayo Clinic

**Keywords:** culture, isolation, Laos, *Rickettsia*, *Orientia*, clinical methods

## Abstract

In the Lao People’s Democratic Republic (Laos), rickettsial infections, including scrub and murine typhus, account for a significant burden of fevers. The Mahosot Hospital Microbiology Laboratory in Vientiane, Laos, routinely performs rickettsial isolation from hospitalized patients with suspected rickettsioses using mammalian cell culture systems. We review the clinical and laboratory factors associated with successful Orientia tsutsugamushi and Rickettsia typhi isolations from this laboratory over a period of 6 years between 2008 and 2014. The overall isolation success was 7.9% for all samples submitted and 17.3% for samples for which the patient had a positive O. tsutsugamushi or R. typhi rapid diagnostic test (RDT), serology, or PCR. The frequency of successful isolation was highest for samples submitted in November, at the end of the wet season (28.3%). A longer median duration of reported illness, a positive result for a concurrent *Orientia* or *Rickettsia* spp. quantitative PCR, and the use of antibiotics by the patient in the week before admission were significantly associated with isolation success (*P* < 0.05). Buffy coat inoculation and a shorter interval between sample collection and inoculation in the laboratory were associated with a higher frequency of isolation (both *P* < 0.05). This frequency was highest if cell culture inoculation occurred on the same day as blood sample collection. Factors related to the initial rickettsial bacterial concentration are likely the main contributors to isolation success. However, modifiable factors do contribute to the rickettsial isolation success, especially delays in inoculating patient samples into culture.

## Abstract (precis)

In the Lao People’s Democratic Republic (Laos), rickettsial infections, including scrub and murine typhus, account for a significant burden of fevers. The Mahosot Hospital Microbiology Laboratory in Vientiane, Laos, routinely performs rickettsial isolation from hospitalized patients with suspected rickettsioses using mammalian cell culture systems. We review the clinical and laboratory factors associated with successful Orientia tsutsugamushi and Rickettsia typhi isolations from this laboratory over a period of 6 years between 2008 and 2014.

## INTRODUCTION

Within the Asia-Pacific region, the rickettsial intracellular bacterial pathogens Orientia tsutsugamushi and Rickettsia typhi are responsible for a significant burden of treatable febrile illnesses. Scrub typhus, caused by O. tsutsugamushi, is the main contributor to this burden and an underrecognized disease affecting predominantly rural areas with poor health infrastructure (1). This disease is transmitted through inoculation by infected chigger mites and is responsible for between 1.8% and 22.3% of fevers among inpatients, depending on region (2, 3), with a median, untreated mortality of approximately 6% (4). Murine typhus, caused by R. typhi, and spotted fever group (SFG) rickettsia are also present within the region. Spread through flea and tick species, these diseases tend to be milder in comparison with scrub typhus, though nonetheless severe disease occurs (5). The epidemiology of murine typhus and SFG are less well characterized but caused 11.4% of febrile illnesses in one case series (6).

Although efficacious treatment exists, clinical diagnosis is difficult due to nonspecific presentations, which are similar to those of other endemic infections, such as malaria and dengue. The presence of an eschar in scrub typhus is variable and diverse presentations, including pneumonitis, septic shock, and meningoencephalitis, are seen across all rickettsial infections (7). When rickettsial infections are suspected, empirical therapy with an agent such as doxycycline is advocated (8). However, this may pose challenges in pregnancy (9) or for infections involving the central nervous system (10).

Current diagnostic strategies are based on quantitative serological testing using immunofluorescence assays (IFA). However, known limitations in IFA performance, the requirements for convalescent-phase sera, and a lack of standardization can make interpretation of serology difficult (11). Other tests, such as enzyme linked immunosorbent assays (ELISAs) and nucleic acid amplification from clinical specimens, can be helpful (12). The *in vitro* isolation of *Orientia* and *Rickettsia* spp. from clinical samples has been practiced for over 60 years. Propagation of organisms allows antibiotic susceptibility testing, genomic analysis, and study of cell-host interactions that are vital for development of vaccines and diagnostic tests (13, 14). However, as culture techniques are insensitive for diagnosis and require prolonged incubation times, their role in clinically useful diagnosis is currently limited (15).

The Lao-Oxford-Mahosot Hospital-Wellcome Trust Research Unit (LOMWRU), based within the Mahosot Hospital Microbiology Laboratory in Vientiane, Lao People’s Democratic Republic (Laos), has been routinely performing rickettsial isolation since 2008. It is currently the only laboratory within Laos equipped for this purpose. Blood samples from febrile patients with suspected rickettsiosis at Mahosot Hospital and regional hospitals in Laos undergo incubation with mammalian cell lines for up to 8 weeks, with the aim of isolating rickettsial organisms. Although there are studies which describe culture conditions used in rickettsial isolation (16), there are none to our knowledge which specifically examine the clinical or laboratory factors associated with successful isolation of O. tsutsugamushi and R. typhi. We therefore undertook a retrospective review of laboratory data at Mahosot Hospital for samples collected for rickettsial culture over 6 years between January 2008 and December 2014.

(A part of this work was presented at the 28th European Congress of Clinical Microbiology and Infectious Diseases, Madrid, Spain, in 2018 and the 2nd Asia Pacific Rickettsia Conference, Chiang Rai, Thailand, in 2019.)

## MATERIALS AND METHODS

All isolation work at LOMWRU was performed in BSL-3 conditions. Up to 5 ml of blood was drawn into EDTA tubes from patients with suspected rickettsioses, all of whom provided written informed consent. Whole EDTA-anticoagulated blood (1 ml) or EDTA buffy coat fraction (200 μl) mixed with 5 ml of RPMI 1640 medium (PAA Laboratories, USA, before 2014 and Gibco, USA, from 2014 onward) and supplemented with 10% fetal calf serum (PAA Laboratories, USA, before 2014 and concurrently with Gibco, USA, from 2014 onward) was used as an inoculum. The buffy coat layer was prepared by centrifugation of EDTA-whole blood at 1,600 × *g* for 10 min. Inoculation was performed directly in 12.5-cm^2^ flasks containing L929 (ATCC number CCL-1) or Vero (ATCC number CCL-81) cell monolayers at 80% confluence, and subsequently centrifuged for 30 min at 50 × *g*. The flask was incubated for 2 h at 35°C in a 5% CO_2_ atmosphere and then the inoculum was washed and replaced with RPMI 1640-10% fetal calf serum medium. Incubation duration was at least eight weeks at 35°C in a 5% CO_2_ atmosphere. Media were changed twice weekly, and subculture was performed at week four by resuspending cells and making a 1:2 or 1:10 dilution into 12.5-cm^2^ or 75-cm^2^ flasks containing L929 or Vero cells at 80% confluence. A scraping of the inoculated cell cultures underwent indirect IFA using pooled antibodies against scrub typhus, spotted fever, and typhus group *Rickettsia* every week after four weeks of incubation or earlier (16). Cultures positive by IFA underwent DNA extraction using DNeasy blood and tissue kit (Qiagen, UK) and growth was confirmed by quantitative PCR (qPCR) targeting the 47-kDa protein or 17-kDa outer membrane antigen (for O. tsutsugamushi and *Rickettsia* spp., respectively) (17). Statistical analyses of patient information and isolation factors were carried out using Stata 14 (Statacorp, USA). Variables were checked as to whether they were normally distributed by the Shapiro-Wilk or Shapiro-Francia tests before using Wilcoxon signed-rank test for nonnormally distributed variables. Median values and interquartile ranges were calculated. Pearson’s chi-squared tests were used to compare frequencies of successful and unsuccessful isolations; exact 95% confidence intervals were calculated for all frequencies. Odds ratios were calculated with 95% confidence intervals and *P* values of ≤0.05 were considered significant. Univariate and multivariate logistical regression was used to determine the significance of variables on culture outcome. Backward multivariate analysis was done, except variables with less than 10 samples were discounted from analysis and variables that returned an odds ratio of 1 during multivariate analysis were also excluded.

### Ethics.

Ethical approvals for collection of samples for determination of the etiology of fever were granted by the Lao National Ethics Committee for Health Research and the Oxford Tropical Research Ethics Committee.

## RESULTS

A total of 3,227 EDTA-anticoagulated whole blood samples were received for rickettsial isolation from 3,200 patients recruited into fever studies conducted in Vientiane (the capital) (*n* = 1,064; 33.0%), Luang Namtha (northern Laos) (*n* = 1,543; 47.8%), and Salavan (southern Laos) (*n* = 620; 19.2%) between January 2008 and December 2014 (see Table S1 in the supplemental material).

Successful isolation was achieved in 256/3,227 (7.9%) samples. O. tsutsugamushi was isolated from 231/256 (90.2%) samples and R. typhi from 24/256 (9.4%) samples, and isolations were confirmed through IFA and qPCR. One blood sample yielded both O. tsutsugamushi and R. typhi, while the species could not be determined for two IFA-positive isolates (0.8%). No spotted fever group rickettsia were cultured. The median duration between incubation start and a positive result was 28 days (interquartile range [IQR]: 23 to 34 days), with no significant difference between organisms cultured (Z = 0.029; *P* = 0.977) or the cell line used (Z = −0.364; *P* = 0.716) (Table S2). Cell culture contamination frequency was 373/6,452 (5.8%). The median age of patients was 26 years (IQR, 16 to 39 years; *n* = 3,213) and was significant between successful and unsuccessful isolations (Z = −2.212; *P* = 0.027). The majority of patients were male, with 45.2% female (see Table 1 and Table S1).

**TABLE 1:** Summary of significant factors in the successful isolation of O. tsutsugamushi and R. typhi

Parameter^*b*^	Successful isolation		Unsuccessful isolation		*P* value
	Frequency (%) or median (*n*)	95% CI or IQR^*c*^	Frequency (%) or median (*n*)	95% CI or IQR^*c*^	
All patients^*a*^					
Median age (yrs)	30 (*n* = 256)	IQR: 18–40.5	26 (*n* = 2,957)	IQR: 16–39	0.027
Median duration of illness (days)	7 (*n* = 253)	IQR: 6–1	7 (*n* = 2,891)	IQR: 4–8	<0.001
Median duration of fever (days)	7 (*n* = 249)	IQR: 5–10	5 (*n* = 2,827)	IQR: 3–7	<0.001
History or presence of:					
Rash	43/254 (16.9%)	CI: 12.5–22.1%	273/2862 (9.5%)	CI: 8.5–10.7%	<0.001
Cough	101/240 (42.1%)	CI: 37.4–50.6%	860/2,348 (36.6%)	CI: 34.7–38.6%	0.029
CNS involvement	5/168 (3.0%)	CI: 1.0–6.8%	98/1,207 (8.1%)	CI: 6.6–9.8%	0.018
Sample type					
Buffy coat	58/291 (19.9%)	CI: 15.5–25.0%			<0.001
EDTA whole blood	193/2,907 (6.6%)	CI: 5.7–7.6%			
Median time between collection and inoculation (days)	2 (*n* = 248)	IQR: 1–3	3 (*n* = 2,766)	IQR: 1–5	<0.001
STG/TG-positive patients^*a*^					
Median duration of illness (days)	7 (*n* = 193)	IQR: 7–10	7 (*n* = 922)	IQR: 5–9	<0.001
History or presence of CNS involvement	4/158 (5.1%)	CI: 0.7–6.4%	75/831 (9.0%)	CI: 7.2–11.2%	0.006
Sample type					
Buffy coat	58/263 (22.1%)	CI: 17.2–27.6%			0.020
EDTA whole blood	133/841 (15.8%)	CI: 13.6–18.7%			
Median time between collection and inoculation (days)	2 (*n* = 191)	IQR: 1–4	3 (*n *= 916)	IQR: 1–7	<0.001

a All patients, the full data set (*n* = 3,227 samples); STG/TG-positive patients, the subset of samples that had either a positive qPCR, positive IgM RDT, and/or a positive IgM or IgG IFA.

b STG, scrub typhus group; TG, typhus group; CNS, central nervous system.

c CI, confidence interval; IQR, interquartile range.

The isolation data set studied was an amalgamation of a number of routine diagnostic and project-based work. In order to provide a more accurate assessment of isolation success, a subset of samples (*n* = 1,126; 34.9%) with other laboratory evidence for rickettsial infection were selected for further analysis. These samples came from patients who had a confirmed positive result for scrub typhus group (STG) or typhus group (TG) rickettsiae by one or more of the following diagnostic tests: (i) Dip-S-Ticks murine typhus RDT (PanBio, now ImmunoDOT Rickettsia typhi, GenBio, adapted to detect IgM) (43.8%); (ii) scrub typhus IgM RDT (Scrub Typhus IgM and IgG Rapid Cassette test, PanBio, Australia; Scrub Typhus Total antibody, AccessBio, USA; CareStart Scrub Typhus IgM Rapid Test Device, AccessBio, USA; or SD Bioline Tsutsugamushi IgM Test, Abbott, USA) (50.0%); (iii) O. tsutsugamushi 47 kDa qPCR (18) (16.0%); (iv) *Rickettsia* spp. 17 kDa qPCR (17) (4.9%); (v) IgG/IgM STG IFA (IgG, 1.2%; IgM, 1.5%); or (vi) IgG/IgM TG IFA (IgG, 2.8%; IgM, 3.3%). IgG and IgM IFA positivity was based on criteria set by studies in Thailand (19, 20) (see Text S1 in the supplemental material) using both admission and convalescent-phase sera or, where not available, only admission sera. Here, these patients are referred to as STG/TG-positive patients, or either STG-positive or TG-positive when referring to patients positive for one particular group of pathogens. The summary of significant factors associated with isolation success is displayed in Table 1 (the full data are provided in Tables S3 to S5).

**FIG 1:**
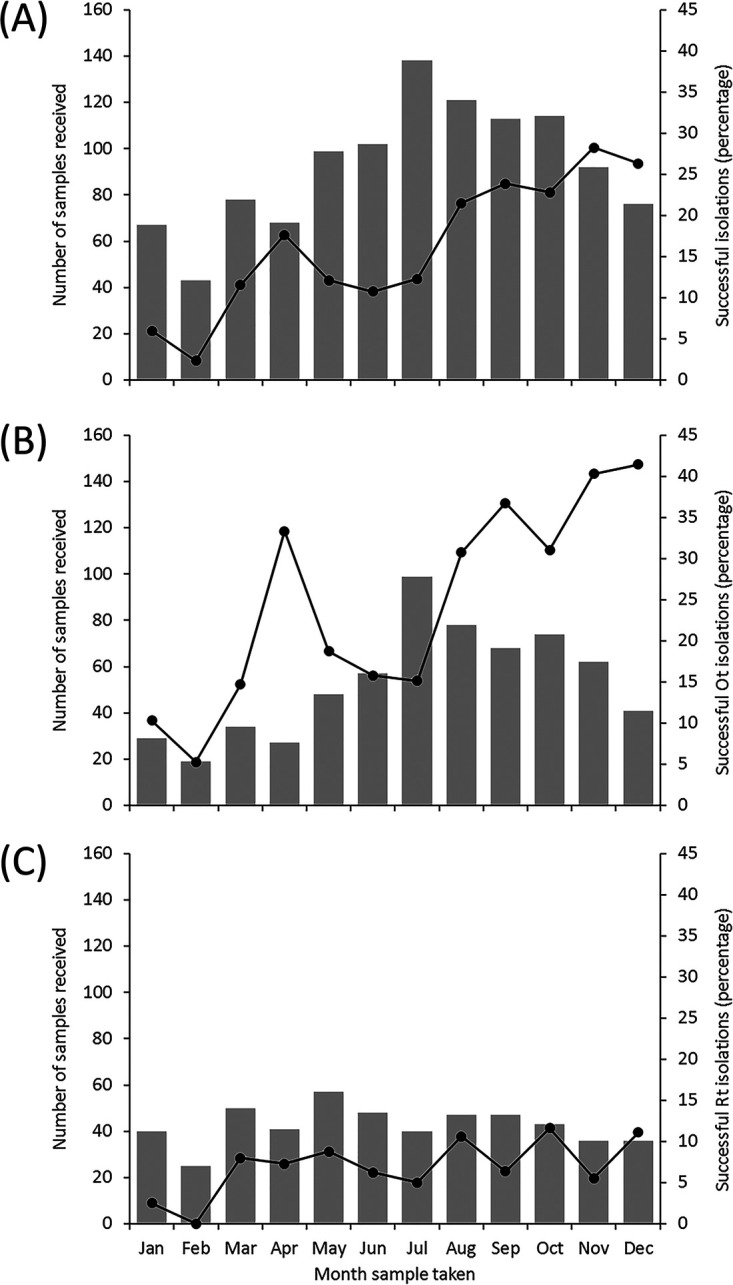
Total number of blood samples submitted for rickettsial isolation (bars) and percentage of those with successful isolations (line) by month between 2008 and 2014. (A) All STG/TG-positive patients (*n* = 1,111). (B) O. tsutsugamushi (Ot) isolations from STG-positive patients (*n* = 636). (C) R. typhi (Rt) isolations from TG-positive patients (*n* = 510).

The frequency of successful isolation from the STG/TG-positive subset was 17.3% (195/1,126), of which 171 were O. tsutsugamushi, 24 were R. typhi, and one isolate was unknown. The month with the greatest number of samples submitted was July (138/1,111, 12.4%), with the fewest in February (43/1,111, 3.9%). However, the frequency of successful isolations was highest from samples submitted in November (26/92, 28.3%) and lowest during February (1/43, 2.3%) (*P* < 0.001) (Fig. 1A). Isolation of O. tsutsugamushi from STG-positive patients was significantly associated with month (*P* < 0.001), with the highest isolation rate seen in December (41.5%) (Fig. 1B), while November had the highest isolation rate for R. typhi from TG-positive patients (11.6%) but with no significant relationship (*P* = 0.804) (Fig. 1C). From all STG/TG-positive patients, successful isolations were significantly associated with a longer duration of illness (*P* < 0.001), a reduced incidence of CNS involvement (i.e., symptoms of confusion, seizures, neck stiffness, or altered consciousness; *P* = 0.006), a history of taking an antibiotic during the previous week (*P* = 0.022), and sample type being buffy coat (*P* = 0.020) (Table 2). In addition to the associations described above, successful isolation of O. tsutsugamushi from STG-positive patients was also associated with a history or presence of a rash (*P* = 0.037) (Table 2 and Table S4). For TG-positive patients, successful isolation of R. typhi was only associated with a history or presence of a cough (*P* = 0.011) or a greater use of antibiotics in the preceding week (*P* < 0.001) (Table 2 and Table S5). The median whole blood volume submitted for isolation was 5.0 ml, but there was no significant association with isolation success (*P* = 0.760) (Table S4). In the 1,006 patients with data, the antibiotic classes prescribed or purchased in the week prior to sampling included beta-lactams (47.3%), tetracyclines (23.8%), quinolones (7.6%), and macrolides (4.4%), with 19.2% of patients taking combination therapy.

**TABLE 2:** Summary of significant factors in the successful isolation of O. tsutsugamushi or R. typhi from samples from STG- or TG-positive patients

Parameter^*b*^	Successful isolation^*a*^		Unsuccessful isolation		*P* value
	Frequency (%) or median (*n*)	95% CI or IQR^*c*^	Frequency (%) or median (*n*)	95% CI or IQR	
STG-positive patients					
Median duration of illness (days)	7 (*n* = 163)	IQR: 7–11	7 (*n* = 486)	IQR: 5–10	0.002
History or presence of rash	26/161 (16.1%)	CI: 10.8–22.8%	48/477 (10.1%)	CI: 7.5–13.1%	0.037
History or presence of CNS involvement	3/129 (2.3%)	CI: 0.5–6.6%	49/421 (11.6%)	CI: 8.7–15.1%	0.002
Sample type					
Buffy coat	45/89 (50.6%)	CI: 39.8–61.3%			<0.001
EDTA whole blood	115/554 (20.8%)	CI: 17.3–24.3%			
Median time between collection and inoculation (days)	2 (*n* = 159)	IQR: 1–4	4 (*n* = 476)	IQR: 2–8	<0.001
TG-positive patients					
History or presence of cough	14/21 (66.6%)	CI: 43.0–85.4%	187/480 (39.0%)	CI: 34.6–43.5%	0.011
Took an antibiotic in the previous wk	10/16 (62.5%)	CI: 35.4–84.8%	151/397 (38.1%)	CI: 33.2–43.0%	<0.001

a Successful isolation of O. tsutsugamushi from STG-positive patients and R. typhi from TG-positive patients.

b STG, scrub typhus group; TG, typhus group; CNS, central nervous system.

c CI, confidence interval; IQR, interquartile range.

Running PCRs (i.e., O. tsutsugamushi 47 kDa qPCR or *Rickettsia* spp. 17 kDa qPCR) on a patient’s EDTA buffy coat sample as a way to select samples for culturing was significantly associated with a higher rate of isolation success for the respective pathogens (65.8% and 38.9%, respectively; *P* < 0.001 for either PCR), although no significance was seen in relation to actual threshold cycle (*C_T_*) values and isolation success (*P* = 0.324 and *P* = 0.709 for O. tsutsugamushi and *Rickettsia* spp., respectively). Similarly, samples from patients with positive IgM IFAs were more likely to have successful isolation for both pathogens (STG = 18.5% success, *P* = 0.026; TG = 60.0% success, *P* < 0.001), while a positive IgG IFA was only significant for R. typhi isolation (88.9% success, *P* < 0.001) (Table 3 and Table S6).

**TABLE 3:** Diagnostic tests that were significantly associated with a successful isolation of O. tsutsugamushi or R. typhi when positive for the target organism

Category	Test(s)^*a*^ (*P* value)
Associated with successful isolation for the whole population	qPCR (<0.001)
Associated with successful isolation of O. tsutsugamushi	O. tsutsugamushi qPCR (<0.001), STG IgM RDT (<0.001), STG IgM IFA (0.026)
Associated with successful isolation of R. typhi	*Rickettsia* spp. qPCR (<0.001), TG IgM RDT (0.001), TG IgM IFA (<0.001), TG IgG IFA (<0.001)

a STG, scrub typhus group; TG, typhus group; IFA, immunofluorescence assay; RDT, rapid diagnostic test; qPCR, quantitative PCR.

**FIG 2:**
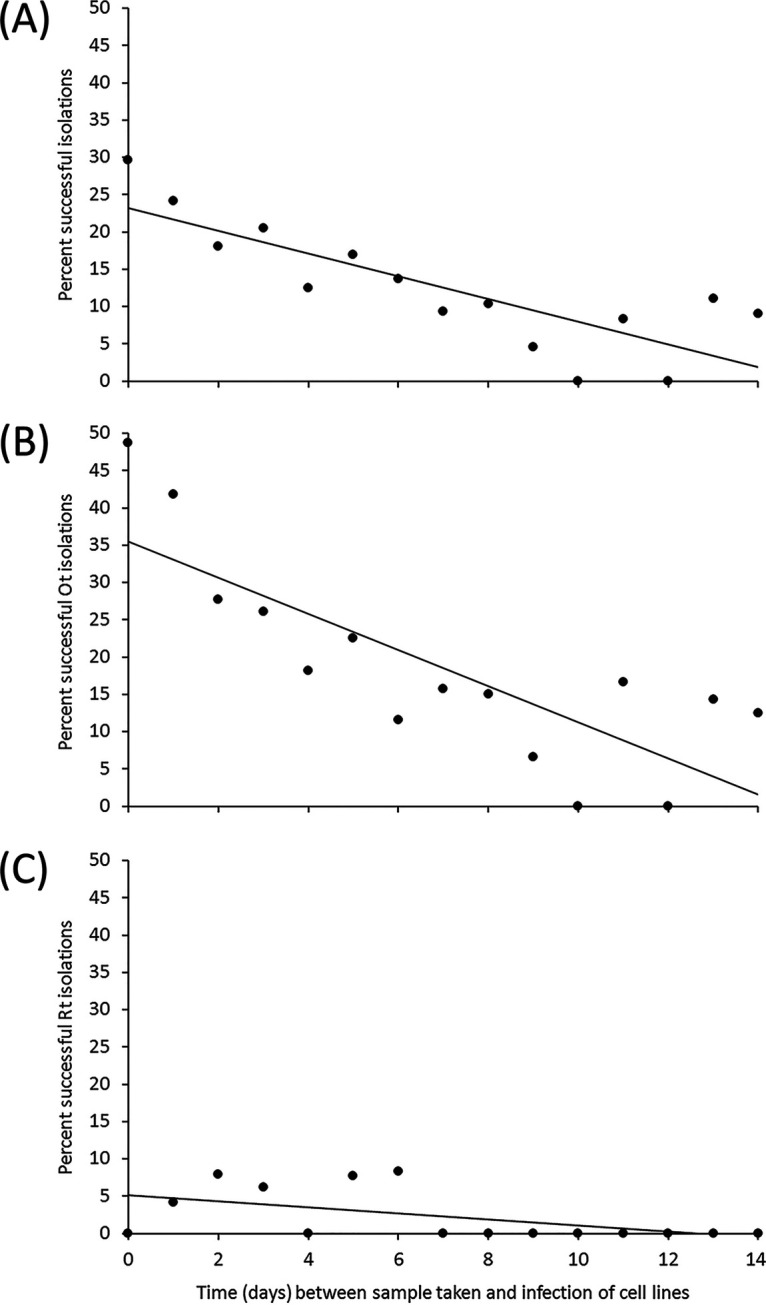
Days between sample collection and inoculation in cell culture versus isolation success. (A) Percentage successful isolation for all STG/TG-positive patients (*R*^2^=0.673; *P* = 0.032). (B) Percentage successful isolation of O. tsutsugamushi from STG-positive patients (*R*^2^=0.640; *P* = 0.252). (C) Percentage successful isolation of R. typhi from TG-positive patients (*R*^2^=0.272, *P* = 0.222).

The majority of blood samples (72.6%) were received by the laboratory for inoculation into cell lines within 5 days. Successful isolation was associated with shorter median interval between blood collection and cell inoculation of 2 days (IQR: 1 to 4) versus 3 days (IQR: 1 to 7) (*n* = 1,107; Z = 5.729, *P* < 0.001) for all STG/TG-positive patients, and 2 days (IQR: 1 to 4) versus 4 days (IQR: 2 to 8) (*n* = 635; Z = 6.922, *P* < 0.001) for all STG-positive patients. For all samples from STG/TG-positive patients, the frequency of successful isolation was highest if the blood sample was inoculated on the same day as collection (29.7%, 19/64) and decreased with samples inoculated on subsequent days (*n* = 938; *P* = 0.032) (Fig. 2). Although not significant, there were also differences between the nine different operators who carried out the isolations, with their overall rates of isolation success ranging from 5.6% to 33.3% (*P* = 0.809).

A multivariate logistic regression model for isolation of either organism from STG/TG-positive patients identified that a positive PCR was significantly associated with isolation success, although the confidence interval was large (odds ratio [OR], 49.3; 95% CI 9.79 to 248.20; *P* < 0.001). Isolation of O. tsutsugamushi from STG-positive patients was significantly associated with a positive O. tsutsugamushi PCR (OR, 9.64; 95% CI 5.42 to 17.16; *P* < 0.001), while the presence of a cough was significant for R. typhi isolation from TG-positive patients (OR, 3.17; 95% CI 1.08 to 9.35; *P* = 0.036) (see Tables S7 to S10).

## DISCUSSION

The *in vitro* isolation of rickettsial organisms from clinical samples is increasingly important in the context of reports of reduced antibiotic susceptibility (21) and the need for understanding the genomic diversity of rickettsial pathogens for development of vaccines and diagnostics.

O. tsutsugamushi was isolated more frequently than R. typhi. Although murine typhus is present in Vientiane (6), it is less common than scrub typhus and therefore this is likely the reason for fewer isolations. In addition, optimal culture conditions may vary between *Rickettsia* spp. (22) and Orientia tsutsugamushi (23) and the cell culture conditions used here may not have been optimal for R. typhi. The optimization of R. typhi isolation should be investigated in greater detail.

The initial rickettsial bacterial load within the blood sample submitted is the likely main predictor of isolation outcome. Overall, *C_T_* values were high (mean = 38.14; IQR = 36.03 to 39.29; *n* = 69), indicating low bacterial loads. Although comparisons between *C_T_* values of successful and unsuccessful isolations do not show any statistical significance, there is a significant relationship between overall qPCR positivity and isolation success in the regression model, as well as a higher isolation frequency through the use of the buffy coat, suggesting an association with bacterial load. The dynamics of rickettsial infection are complex. An early, limited bacteremic phase for O. tsutsugamushi before intracellular entry has been described from animal models (24), consistent with a relatively low median number of organisms (<100 copies/μl) within human blood (25, 26). Within our cohort, a longer duration of illness was associated with a successful isolation, probably because prolonged illness is associated with a higher bacterial burden. The severity of illness has been associated with higher bacterial burdens in O. tsutsugamushi and R. typhi infections (27) and potentially why we see that the presence of a cough (for R. typhi) or a rash (for O. tsutsugamushi) is significantly associated with successful isolation. We also found a significant association between isolation and antibiotic use in the preceding week before admission, although, unexpectedly, this was related to an increase in successful isolations. One possible explanation for this is that patients with more severe disease had a higher chance of seeking antibiotics, but the majority (74%) of those antibiotics taken would not be efficacious against either rickettsial pathogen (28, 29).

The use of PCR to screen for possible positive patients showed a significant association with isolation success when positive. This may be expected, as PCRs rely on the detection of bacterial DNA in the sample, indicative of a current infection. IFAs were not used to select samples for isolation in any of the associated studies here and were completed after samples had been sent for isolation. As would be expected, positive IgM IFAs are significantly associated with a successful isolation, as IgM is indicative of a current or recent infection. Although positive RDTs are also significantly associated with successful isolations (see Table S6 in the supplemental material), the majority of attempted isolations were carried out based on positive RDT results; therefore, it is difficult to interpret their usefulness without discounting the risk of bias in the data. This information highlights the benefit of using diagnostic tests to guide the selection of samples for isolation.

Temporal changes in isolation success for O. tsutsugamushi reflect the observed seasonality of scrub typhus, which appears after onset of the annual monsoon season (6, 8). Isolation success frequency was highest for blood samples collected at the end of the wet season (28.3% for all rickettsial pathogens) and lowest for those submitted in the middle of the dry season (2.3%). The seasonal increase in isolation rate during the wet season may result through increased caseload from chigger exposure in activities such as rice harvest, as well as ecological factors which tend to increase vector density and alter feeding behavior during that period (30). A similar pattern, although on a much smaller scale, is seen with R. typhi isolation. Although not statistically significant, seasonal changes are most likely due to the same factors because seasonality in regional flea abundance has been previously identified (31).

A shorter time between blood collection and inoculation was associated with higher rates of isolation and may relate to decline in viability of the bacteria in *ex vivo* blood with time. Delays occur because samples collected from study sites have to be transported to the laboratory in Vientiane, and rickettsial isolation was not performed during weekends. The impact of delayed inoculation has been demonstrated for R. conorii, for which the frequency of isolation through shell-vial culture was reduced when clinical samples were not inoculated on the same day as blood draw (32). Systems aimed to shorten the time to inoculation or maintain viability though use of hemoculture fluid (33) may improve isolation success; this is important as the initial bacterial concentration in clinical samples is likely to be low. We also found variation in success rates between different technicians performing the isolation, most likely due to an individual’s level of training and experience. Further evaluation or competency training, along with routine audit, would be essential for standardizing practices. The findings within the multivariate model that PCR was the most significant factor at predicting isolation success suggest that current culture success depends largely on initial patient and bacterial factors. Laboratory data, in respect to isolations, were complete and 3,145/3,227 (97.5%) patients had at least some clinical information available, although this was highly variable. Prehospital antibiotic use data were only available for 1,767/3,200 (55.2%) patients and qPCR data available for 1,610/3,227 samples (49.9%).

In summary, we describe the clinical and laboratory factors associated with O. tsutsugamushi and R. typhi isolation success in Laos. Similar to the culture of conventional bacteria from blood, we found that host factors such as illness duration and factors indicative of initial blood bacterial concentration are strongly associated with isolation success. It will be important to further define the role of culture technique to aid staff training and reduce delayed sample inoculation to improve the isolation yield and support research in this group of diseases.

## Supplementary Material

Supplemental file 1

Supplemental file 2
