# Supplementary material for: The Isolation of Orientia tsutsugamushi and Rickettsia typhi from Human Blood through Mammalian Cell Culture: a Descriptive Series of 3,227 Samples and Outcomes in the Lao People’s Democratic Republic
Source: J Clin Microbiol. 2020 Nov 18;58(12):e01553-20. doi: 10.1128/JCM.01553-20 (PMC7685894; doi:10.1128/JCM.01553-20)
Supplement: Supplemental file 1 [file JCM.01553-20-s0001.pdf]

Supplementary information Criteria for IFA results

Identification of positive and negative IgM and IgG IFA results was made using the following criteria:

Scrub typhus (STG) (based on Lim *et al*, 2015):

IgM: ≥1:3,200 at admission and/or ≥1:3,200 at follow-up with 4-fold rise compared to admission.

IgG: ≥1:1,600 at admission and/or ≥1:1,600 at follow-up with 4-fold rise compared to admission.

Murine typhus (TG) (based on Phakhounthong *et al*. 2019):

IgM: ≥1:800 at admission and/or ≥1:800 at follow-up with 4-fold rise compared to admission.

IgG: ≥1:1,600 at admission and/or ≥1:1,600 at follow-up with 4-fold rise compared to admission

References

Lim C, Blacksell SD, Laongnualpanich A, Kantipong P, Day NP, Paris DH, Limmathurotsakul D. 2015. Optimal cutoff titers for indirect immunofluorescence assay for diagnosis of scrub typhus. J Clin Microbiol 53:3663-6.

Phakhounthong K, Mukaka M, Dittrich S, Tanganuchitcharnchai A, Day NPJ, White LJ, Newton PN, Blacksell SD. 2020. The temporal dynamics of humoral immunity to *Rickettsia typhi* infection in murine typhus patients. Clin Microbiol Infect 26:781 e9-781 e16.
