# Supplementary material for: The Isolation of Orientia tsutsugamushi and Rickettsia typhi from Human Blood through Mammalian Cell Culture: a Descriptive Series of 3,227 Samples and Outcomes in the Lao People’s Democratic Republic
Source: J Clin Microbiol. 2020 Nov 18;58(12):e01553-20. doi: 10.1128/JCM.01553-20 (PMC7685894; doi:10.1128/JCM.01553-20)
Supplement: Supplemental file 2 [file JCM.01553-20-s0002.pdf]

TABLE S1 Patient and laboratory characteristics between successful and unsuccessful *O. tsutsugamushi* and *R. typhi* isolations for all attempted isolations.

	All		*Successful isolation*		*Unsuccessful isolation*			
	Frequency (%) or median (n)	*95% CI or IQR*[a]	*Frequency (%) or median (n)*	*95% CI or IQR*[a]	*Frequency (%) or median (n)*	*95% CI or IQR*[a]	*z score*	*p-value*[b]
Number of patients	n=3,200		255/3,200 (8.0%)	CI: 7.1-9.0%				
Number of samples	n=3,227		256/3,227 (7.9%)	CI: 7.0-8.9%				
Successful isolations								
O. tsutsugamushi			231/256 (90.2%)	CI: 85.9-936%				
R. typhi			24/256 (9.4%)	CI: 6.1-13.6%				
Unknown			2/256 (0.8%)	CI: 0.1-2.8%				
Location								
Vientiane	1,064/3,227 (33.0%)	CI: 31.4-34.6%	166/256 (64.8%)	CI: 58.7-70.7%				
Luang Namtha	1,543/3,227 (47.8%)	CI: 46.1-49.6%	66/256 (25.8%)	CI: 20.5-31.6%				
Saravan	620/3,227 (19.2%)	CI: 17.9-20.6%	24/256 (9.4%)	CI: 6.1-13.6%				
Median age (years)	26 (n=3,213)	IQR: 16-39	30 (n=256)	IQR: 18-40.5	26 (n=2,957)	IQR: 16-39	-2.212	0.027
Sex (female)	1,456/3,224 (45.2%)	CI: 43.4-46.9%	115/256 (44.9%)	CI: 38.7-51.2%	1,341/2,968 (45.2%)	CI: 43.4-47.0%		0.936
Median duration of illness (days)	7 (n=3,144)	IQR: 5-8	7 (n=253)	IQR: 6-1	7 (n=2,891)	IQR: 4-8	-7.537	**<0.001**
Median duration of fever (days)	6 (n=3,076)	IQR: 4-8	7 (n=249)	IQR: 5-10	5 (n=2,827)	IQR: 3-7	-4.861	**<0.001**
History or presence of…								
…rash	316/3,116 (10.1%)	CI: 9.1-11.3%	43/254 (16.9%)	CI: 12.5-22.1%	273/2,862 (9.5%)	CI: 8.5-10.7%		**<0.001**
…cough	961/2,578 (37.3%)	CI: 35.4-39.2%	101/240 (42.1%)	CI: 37.4-50.6%	860/2,348 (36.6%)	CI: 34.7-38.6%		**0.029**
…headache	2,806/3,099 (90.6%)	CI: 89.5-91.6%	230/250 (92.0%)	CI: 87.9-95.0%	2,576/2,849 (90.4%)	CI: 89.3-91.5%		0.412
…CNS involvement[c]	103/1,375 (7.5%)	CI: 6.-9.0%	5/168 (3.0%)	CI: 1.0-6.8%	98/1,207 (8.1%)	CI: 6.6-9.8%		**0.018**

…fever	3,111/3,145 (98.9%)	CI: 98.5-99.3%	251/254 (98.8%)	CI: 96.6-99.8%	2,860/2,891 (98.9%)	CI: 98.5-99.3%		0.872
Took an antibiotic in the previous week?	1,127/1,778 (63.4%)	CI: 61.1-65.6%	117/187 (62.6%)	CI: 55.2-69.5%	1,010/1,591 (63.5%)	CI: 61.1-65.9%		0.166
Sample type								
Buffy coat			58/291 (19.9%)	CI: 15.5-25.0%				**<0.001**
EDTA whole blood			193/2,907 (6.6%)	CI: 5.7-7.6%				
Median time between collection and inoculation (days)	3 (n=3,014)	IQR: 1-5	2 (n=248)	IQR: 1-3	3 (n=2,766)	IQR: 1-5	5.072	**<0.001**
Median sample volume (mls EDTA whole blood)	4 (n=2,407)	IQR: 3-5	4 (n=146)	IQR: 3-5	4 (n=2,261)	IQR: 3-5	-1.723	0.085

[a]CI = confidence interval, IQR = Interquartile range; [b]significant p-values shown in bold; [c]CNS involvement determined by any one feature of confusion, seizure, neck stiffness or altered consciousness.

Table S2 Time taken for a successful isolation to be identified as positive from day of inoculation of cell lines.

Factor	All successful isolations	Pathogen isolated				Cell line used			
		Rt isolated[a]	*Ot* isolated[a]	*z score*	*p-value*	Vero	L929	*z score*	*p-value*
Days until positive, median (n), IQR[b]	28 (n=367) IQR: 23-34	27 (n=33) IQR: 23-37	28 (n=333) IQR: 23-34	0.029	0.977	28 (n=209) IQR: 23-34)	28 (n=158) IQR: 23-34	-0.364	0.716

[a]*Rt* = *R. typhi* and *Ot* = *O. tsutsugamushi*; [b]IQR = Interquartile range.

Table S3 Patient and laboratory characteristics between successful and unsuccessful *O. tsutsugamushi* and *R. typhi* isolations from STG/TG-positive patients.

	All		*Successful isolation*		*Unsuccessful isolation*			
	Frequency (%) or median (n)	*95% CI or IQR*[a]	*Frequency (%) or median (n)*	*95% CI or IQR*[a]	*Frequency (%) or median (n)*	*95% CI or IQR*[a]	*z score*	*p-value*[b]
Number of patients	n=1107		194/1,107 (17.5%)	CI: 15.3-19.9%				
Number of samples	n=1126		195/1,126 (17.3%)	CI: 15.2-19.7%				
Successful isolations								
O. tsutsugamushi			171/195 (87.7%)	CI: 82.2-92.0%				
R. typhi			24/195 (12.3%)	CI: 8.0-17.8%				
Unknown			1/195 (0.5%)	CI: 0.01-2.8%				
Location								
Vientiane (n)	1,037/1,126 (92.1%)	CI: 90.4-93.6%	165/195 (84.6%)	CI: 78.8-89.4%				
Luang Namtha (n)	57/1,126 (5.1%)	CI: 3.9-6.5%	22/195 (11.3%)	CI: 7.2-16.6%				
Saravan (n)	32/1,126 (2.8%)	CI: 2.0-4.0%	8/195 (4.1%)	CI: 1.8-7.9%				
Median age (years)	30 (n=1,126)	IQR: 21-43	32 (n=195)	IQR: 20-43	30 (n=931)	IQR: 21-43	-0.049	0.961
Sex (female)	516/1,126 (45.8%)	CI: 42.9-48.8%	88/195 (45.1%)	CI: 38.0-52.4%	428/931 (46.0%)	CI: 42.7-49.2%		0.830
Median duration of illness (days)	7 (n=1,115)	IQR: 5-9	7 (n=193)	IQR: 7-10	7 (n=922)	IQR: 5-9	-3.602	**<0.001**
Median duration of fever (days)	7 (n=1,046)	IQR: 4-9	7 (n=189)	IQR: 4-9	7 (n=857)	IQR: 4-9	-0.076	0.939
History or presence of…								
…rash	159/1,103 (14.4%)	CI: 12.4-16.6%	31/193 (16.1%)	CI: 11.2-22.0%	128/910 (14.1%)	CI: 11.9-16.5%		0.473
…cough	429/1,077 (39.8%)	CI: 36.9-42.8%	80/185 (43.2%)	CI: 36.0-50.7%	349/892 (39.1%)	CI: 35.9-42.4%		0.298
…headache	1,007/1,107 (91.0%)	CI: 89.1-92.6%	173/189 (91.5%)	CI: 86.6-95.1%	834/918 (90.9%)	CI: 88.8-92.6%		0.765
…CNS involvement[c]	79/989 (8.0%)	CI: 6.4-9.9%	4/158 (5.1%)	CI: 0.7-6.4%	75/831 (9.0%)	CI: 7.2-11.2%		**0.006**

…fever	1,087/1,105 (98.4%)	CI: 97.4-99.0%	190/193 (98.5%)	CI: 95.5-99.7%	897/912 (98.4%)	CI: 97.3-99.1%		0.928
Took an antibiotic in the previous week?	412/903 (45.6%)	CI: 42.3-48.9%	87/156 (55.8%)	CI: 47.6-63.7%	325/747 (43.5%)	CI: 39.9-47.1%		**0.022**
Sample type								
Buffy coat			58/263 (22.1%)	CI: 17.2-27.6%				**0.020**
EDTA whole blood			133/841 (15.8%)	CI: 13.6-18.7%				
Median time between collection and inoculation (days)	3 (n=1,107)	IQR: 1-6	2 (n=191)	IQR: 1-4	3 (n=916)	IQR: 1-7	5.729	**<0.001**
Median sample volume (mls EDTA whole blood)	5 (n=465)	IQR: 4-5	5 (n=88)	IQR: 3.75-5	5 (n=377)	IQR: 4-5	1.373	0.170

[a]CI = confidence interval, IQR = Interquartile range; [b]significant p-values shown in bold; [c]CNS involvement determined by any one feature of confusion, seizure, neck stiffness or altered consciousness.

Table S4 Patient and laboratory characteristics between successful and unsuccessful *O. tsutsugamushi* (*Ot*) isolations from STG-positive patients.

	All STG-positive		*Successful Ot isolation*		*Unsuccessful isolation*			
	Frequency (%) or median (n)	*95% CI or IQR[a]*	*Frequency (%) or median (n)*	*95% CI or IQR[a]*	*Frequency (%) or median (n)*	*95% CI or IQR[a]*	*z score*	*p-value[b]*
Number of patients	645		162/645 (26.1%)	CI: 21.8-28.6%				
Number of samples	649		163/649 (25.1%)	CI: 21.8-28.6%				
Median age (years)	30 (n=649)	IQR: 19-42	31 (n=163)	IQR: 18-42	30 (n=486)	IQR: 19-43	-0.226	0.821
Sex (female)	273/649 (42.1%)	CI: 38.2-46.0%	76/136 (46.6%)	CI: 38.8-54.6%	197/486 (40.5%)	CI: 36.1-45.0%		0.173
Median duration of illness (days)	7 (n=644)	IQR: 5-10	7 (n=161)	IQR: 7-11	7 (n=483)	IQR: 5-10	-0.991	**0.002**
Median duration of fever (days)	7 (n=636)	IQR: 6-9	7 (n=159)	IQR: 7-8	7 (n=477)	IQR: 6-9	-0.792	0.428
History or presence of…								
…rash	74/638 (11.6%)	CI: 9.2-14.3%	26/161 (16.1%)	CI: 10.8-22.8%	48/477 (10.1%)	CI: 7.5-13.1%		**0.037**
…cough	243/611 (39.8%)	CI: 35.9-43.8%	62/153 (40.5%)	CI: 32.7-48%	181/458 (39.5%)	CI: 35.0-44.2%		0.826
…headache	573/637 (90.0%)	CI: 87.3-92.2%	143/157 (91.1%)	CI: 85.5-95.0%	429/479 (89.6%)	CI: 86.5-92.2%		0.582
…CNS involvement[c]	52/550 (9.5%)	CI: 7.1-12.2%	3/129 (2.3%)	CI: 0.5-6.6%	49/421 (11.6%)	CI: 8.7-15.1%		**0.002**
…fever	632/643 (98.3%)	CI: 97.0-99.1%	158/161 (98.1%)	CI: 94.7-99.6%	474/482 (98.3%)	CI: 96.8-99.3%		0.863
Took an antibiotic in the previous week?	262/521 (50.3%)	CI: 45.9-54.7%	72/131 (55.0%)	CI: 46.0-63.7%	190/390 (48.7%)	CI: 43.7-53.8%		0.393
Sample type								
Buffy coat			45/89 (50.6%)	CI: 39.8-61.3%				**<0.001**
EDTA whole blood			115/554 (20.8%)	CI: 17.3-24.3%				
Median time between collection and inoculation (days)	3 (n=635)	IQR: 1-6	2 (n=159)	IQR: 1-4	4 (n=476)	IQR: 2-8	6.922	<0.001
Median sample volume (mls EDTA whole blood)	5 (n=309)	IQR: 4-5	5 (n=77)	IQR: 3.5-5	5 (n=232)	IQR: 4-5	0.705	0.481

[a]CI = confidence interval, IQR = Interquartile range; [b]significant p-values shown in bold; [c]CNS involvement determined by any one feature of confusion, seizure, neck stiffness or altered consciousness.

TABLE S5 Patient and laboratory characteristics between successful and unsuccessful *R. typhi* (*Rt*) isolations from TG-positive patients.

	All TG-positive		*Successful Rt isolation*		*Unsuccessful isolation*			
	Frequency (%) or median (n)	*95% CI or IQR*[a]	*Frequency (%) or median (n)*	*95% CI or IQR*[a]	*Frequency (%) or median (n)*	*95% CI or IQR*[a]	*z score*	*p-value*[b]
Number of patients	n=496		21/496 (4.2%)	CI: 2.6-6.4%				
Number of samples	n=512		21/512 (4.1%)	CI: 2.6-6.2%				
Median age (years)	32 (n=512)	IQR: 23.5-45	45 (n=21)	IQR: 30-56	32 (n=491)	IQR: 23-44	-1.877	0.061
Sex (female)	254/512 (49.6%)	CI: 45.2-54.0%	7/21 (33.3%)	CI: 14.6-57.0%	247/491 (50.3%)	CI: 45.8-54.8%		0.128
Median duration of illness (days)	7 (n=506)	IQR: 5-9	7 (n=21)	IQR: 6-9	7 (n=485)	IQR: 5-9	-3.077	0.322
Median duration of fever (days)	7 (n=443)	IQR: 1-9	7 (n=20)	IQR: 1-9	7 (n=423)	IQR: 1-9	0.022	0.983
History or presence of…								
…rash	91/499 (18.2%)	CI: 14.9-21.9%	3/21 (14.3%)	CI: 3.0-36.3%	88/478 (18.4%)	CI: 15.0-22.2%		0.632
…cough	201/501 (40.1%)	CI: 35.8-44.6%	14/21 (66.6%)	CI: 43.0-85.4%	187/480 (39.0%)	CI: 34.6-43.5%		**0.011**
…headache	468/506 (92.5%)	CI: 89.9-94.6%	20/21 (95.2%)	CI: 76.2-99.9%	448/485 (92.4%)	CI: 89.7-94.6%		0.626
…CNS involvement[c]	31/470 (6.6%)	CI: 4.5-9.2%	1/19 (5.0%)	CI: 0.1-24.9%	30/420 (6.7%)	CI: 4.5-9.4%		0.769
…fever	488/497 (98.1%)	CI: 96.6-99.2%	21/21 (100.0%)	CI: 83.9-100%[d]	467/476 (98.1%)	CI: 96.4-99.1%		0.053
Took an antibiotic in the previous week?	161/413 (39.0%)	CI: 34.3-43.9%	10/16 (62.5%)	CI: 35.4-84.8%	151/397 (38.1%)	CI: 33.2-43.0%		**<0.001**
Sample type								
Buffy coat			11/189 (5.8%)	CI: 2.9-10.2%				0.112
EDTA whole blood			9/307 (2.9%)	CI: 1.5%-6.0%				
Median time between collection and inoculation (days)	2 (n=507)	IQR: 1-5	2 (n=21)	IQR: 1-3	2 (n=486)	IQR: 1-5	0.897	0.370
Median sample volume (mls EDTA whole blood)	5 (n=166)	IQR: 5-5	5 (n=7)	IQR: 3-5	5 (n=159)	IQR: 3-5	0.435	0.663

[a]CI = confidence interval, IQR = Interquartile range; [b]significant p-values shown in bold; [c]CNS involvement determined by any one feature of confusion, seizure, neck stiffness or altered consciousness; [d]one-sided, 97.5% confidence interval.

TABLE S6 Association of isolation with diagnostic test performed on patient[a].

		All		*Successful isolation*		*Unsuccessful isolation*		
		Frequency (%)	95% CI	Frequency (%)	95% CI	Frequency (%)	95% CI	*p-value*[b]
Whole population	Positive PCR	234/1,610 (14.5%)	12.8-16.4%	88/138 (63.8%)	55.2-71.8%	146/1,472 (9.9%)	8.4-11.6%	**<0.001**
	Positive IgM RDT	1,035/1,066 (97.1%)	95.9-98.0%	165/166 (99.4%)	96.7-100.0%	870/900 (96.7%)	95.3-97.7%	0.054
	Positive IgM IFA	53/268 (19.8%)	15.2-25.1%	11/42 (26.2%)	13.9-42.0%	42/226 (18.6%)	13.7-24.3%	0.256
	Positive IgG IFA	44/213 (20.7%)	15.4-26.7%	10/36 (27.8%)	14.2-45.2%	34/177 (19.2%)	13.7-25.8%	0.247
STG-specific tests with *Ot* isolation	Positive PCR			79/120 (65.8%)	56.6-74.2%	101/1,487 (6.8%)	5.6-8.2%	**<0.001**
	Positive IgM RDT			132/140 (94.3%)	89.1-97.5%	431/887 (48.6%)	45.3-51.9%	**<0.001**
	Positive IgM IFA			5/27 (18.5%)	6.3-38.1%	12/191 (6.3%)	3.3-10.7%	**0.026**
	Positive IgG IFA			2/24 (8.3%)	1.0-27.0%	12/134 (9.0%)	4.7-15.1%	0.921
TG-specific tests with *Rt* isolation	Positive PCR			7/18 (38.9%)	17.3-64.3%	48/1,590 (3.0%)	2.2-4.0%	**<0.001**
	Positive IgM RDT			21/21 (100.0%)	83.9-100.0%[c]	472/747 (63.2%)	59.6-66.7%	**0.001**
	Positive IgM IFA			6/10 (60.0%)	26.2-87.8%	31/229 (13.5%)	9.4-18.7%	**<0.001**
	Positive IgG IFA			8/9 (88.9%)	51.8-99.1%	23/167 (13.8%)	8.9-19.9%	**<0.001**

[a]STG = Scrub typhus Group, TG = Typhus Group, *Rt* = *R. typhi*, *Ot* = *O. tsutsugamushi;* [b]significant p-values shown in bold; [c]one-sided, 97.5% confidence interval.

Table S7 Univariate and multivariate logistical regression of whole population of samples submitted for isolation. Reference is success of culturing *R. typhi* or *O. tsutsugamushi*.

Factor	Number of samples in agreement	Number of samples in agreement, with successful isolation	Logistical regression model			
			Univariate analysis		Multivariate analysis[a]	
			OR (95% CI)	*p-value*[b]	OR (95% CI)	*p-value*[b]
Sex (Female)	1,456/3,224	115	0.99 (0.77-1.28)	0.936	0.59 (0.17-2.04)	0.406
Fever (Yes)	3,111/3,145	251	0.91 (0.28-2.99)	0.872	-	-
Cough (Yes)	961/2,578	101	1.35 (1.03-1.78)	**0.030**	0.45 (0.12-1.66)	0.229
Headache (Yes)	2,806/3,099	230	1.22 (0.76-0.96)	0.413	0.48 (0.03-8.85)	0.618
Rash (Yes)	316/3,116	43	1.93(1.36-2.74)	**<0.001**	0.65 (0.12-3.44)	0.609
CNS involvement (Yes)	103/1,375	5	0.35 (0.14-0.87)	0.023	-	-
Antibiotic last week (Yes)	1,127/1,778	117	0.96 (0.70-1.32)	0.806	1.64 (0.50-5.36)	0.412
qPCR (Positive)	234/1,610	88	15.98 (10.86-23.54)	**<0.001**	50.20 (9.96-253.13)	**<0.001**
RDT (IgM) (Positive)	1,035/1,066	165	5.69 (0.77-42.01)	0.088	-	-
IFA (IgM) (Positive)	53/268	11	1.55 (0.72-3.34)	0.258	1.43 (0.22-9.26)	0.706
IFA (IgG) (Positive)	44/213	10	1.62 (0.71-3.67)	0.250	0.83 (0.11-6.17)	0.853

[a]For multivariate analysis, factors with less than 10 samples were discounted from analysis. Factors that returned an Odds Ratio of 1 during multivariate analysis were excluded; [b]significant *p-values* shown in bold.

Table S8 Univariate and multivariate logistical regression of samples from STG/TG-positive patients submitted for isolation. Reference is success of culturing *R. typhi* or *O. tsutsugamushi*.

Factor	Number of samples in agreement	Number of samples in agreement, with successful isolation	Logistical regression model			
			Univariate analysis		Multivariate analysis[a]	
			OR (95% CI)	*p-value*[b]	OR (95% CI)	*p-value*[b]
Sex (Female)	516/1,126	88	0.97 (0.71-1.32)	0.830	0.60 (0.17-2.06)	0.416
Fever (Yes)	1,087/1,105	190	1.06 (0.30-3.69)	0.928	-	-
Cough (Yes)	429/1,077	80	1.19 (0.86-1.63)	0.298	0.44 (0.12-1.65)	0.226
Headache (Yes)	1,007/1,107	173	1.09 (0.62-1.90)	0.765	0.48 (0.03-8.87)	0.623
Rash (Yes)	159/1,103	31	1.17 (0.76-1.79)	0.474	0.64 (0.12-3.42)	0.602
CNS involvement (Yes)	79/989	4	0.26 (0.09-0.73)	**0.010**	**-**	-
Antibiotic last week (Yes)	412/903	87	1.64 (1.16-2.32)	**0.005**	1.65 (0.51-5.40)	0.406
qPCR (Positive)	234/770	88	7.09 (4.70-10.70)	<**0.001**	49.30 (9.79-248.20)	<**0.001**
RDT (IgM) (Positive)	1,035/1,037	165	1	n/a	-	-
IFA (IgM) (Positive)	53/260	11	1.49 (0.69-3.20)	0.310	1.41 (0.22-9.20)	0.719
IFA (IgG) (Positive)	41/204	10	1.70 (0.74-3.89)	0.209	0.84 (0.11-6.40)	0.869

[a]For multivariate analysis, factors with less than 10 samples, or which had an Odds Ratio of 1 in univariate analysis were discounted from analysis. Factors that returned an Odds Ratio of 1 during multivariate analysis were excluded; [b]significant *p-values* shown in bold.

Table S9 Univariate and multivariate logistical regression of samples from patients with a positive diagnosis for STG submitted for isolation of *O. tsutsugamushi* (*Ot*). Reference is success of culturing *O. tsutsugamushi*.

Factor	Number of samples in agreement	Number of samples in agreement, with successful *Ot* isolation	Logistical regression model			
			Univariate analysis		Multivariate analysis[a]	
			OR (95% CI)	*p-value*[b]	OR (95% CI)	*p-value*[b]
Sex (Female)	273/649	76	1.28 (0.90-1.83)	0.173	1.46 (0.82-2.59)	0.199
Fever (Yes)	632/643	158	0.89 (0.23-3.39)	0.863	-	-
Cough (Yes)	243/611	62	1.04 (0.72-1.51)	0.826	0.83 (0.46-1.50)	0.541
Headache (Yes)	572/636	143	1.19 (0.64-2.22)	0.583	2.22 (0.68-7.29)	0.189
Rash (Yes)	74/638	26	1.72 (1.03-2.88)	**0.039**	1.9 (0.87-4.16)	0.106
CNS involvement (Yes)	52/550	3	0.18 (0.06-0.59)	**0.005**	-	-
Antibiotic last week (Yes)	262/521	72	1.28 (0.86-1.91)	0.217	0.9 (0.50-1.60)	0.716
O. tsutsugamushi qPCR (Positive)	180/488	79	7.25 (4.49-11.69)	**<0.001**	9.64 (5.42-17.16)	**<0.001**
STG RDT (IgM) (Positive)	563/566	132	1	n/a	-	-
STG RDT (IgG) (Positive)	2/5	1	1	n/a	-	-
STG IFA (IgM) (Positive)	17/141	5	1	n/a	-	-
STG IFA (IgG) (Positive)	10/107	2	1	n/a	-	-

[a]For multivariate analysis, factors with less than 10 samples, or which had an Odds Ratio of 1 in univariate analysis were discounted from analysis. Factors that returned an Odds Ratio of 1 during multivariate analysis were excluded; [b]significant *p-values* shown in bold.

Table S10 Univariate and multivariate logistical regression of samples from patients with a positive diagnosis for TG submitted for isolation of *R. typhi* (*Rt*). Reference is success of culturing *R. typhi*.

Factor	Number of samples in agreement	Number of samples in agreement, with successful *Rt* isolation	Logistical regression model			
			Univariate analysis		Multivariate analysis[a]	
			OR (95% CI)	*p-value*[b]	OR (95% CI)	*p-value*[b]
Sex (Female)	254/512	7	0.49 (0.20-1.24)	0.135	-	-
Fever (Yes)	488/497	21	1	n/a	-	-
Cough (Yes)	201/501	14	3.13 (1.24-7.91)	**0.016**	3.17 (1.08-9.35)	**0.036**
Headache (Yes)	468/506	20	1.65 (0.22-12.65)	0.629	1.36 (0.17-10.89)	0.775
Rash (Yes)	91/499	3	0.74 (0.21-2.56)	0.633	-	-
CNS involvement (Yes)	31/470	1	0.74 (0.10-5.69)	0.770	-	-
Antibiotic last week (Yes)	161/413	10	2.72 (0.97-7.62)	0.058	2.63 (0.93-7.44)	0.069
Rickettsia spp. qPCR (Positive)	55/300	7	4.32 (1.50-12.48)	**0.007**	**-**	-
TG RDT (IgM) (Positive)	493/494	21	1	n/a	-	-
TG IFA (IgM) (Positive)	37/124	6	4.02 (1.06-15.20)	**0.041**	**-**	-
TG IFA (IgG) (Positive)	29/103	8	27.81 (3.29-235.13)	**0.002**	-	-

[a]For multivariate analysis, factors with less than 10 samples, or which had an Odds Ratio of 1 in univariate analysis were discounted from analysis. Factors that returned an Odds Ratio of 1 during multivariate analysis were excluded; [b]significant *p-values* shown in bold.
